# The International Medical Congress

**Published:** 1886-11

**Authors:** 


### THE INTERNATIONAL MEDICAL CONGRESS.

The Executive Committee of the Congress, to whom has been entrusted the work of organizing, met in Pittsburgh on October 11. The meeting was fully attended, and the various reports of the sub-committees and the extensive correspondence of its chairman all showed the advance made in the organization of the Congress. The presidents of all of the Sections of the Congress reported highly satisfactory progress in the appointment of sectional officers, and promises of original papers of a high class. The various officers of the Sections, including members of the Council, now exceed three hundred and seventy-five well-known members of the medical profession in the United States. Numerous appointments to Sections have also been accepted by prominent members of the profession in Great Britain, France, Belgium, Holland, Germany, Brazil, and other countries; so that there can now be no doubt as to the international character of the Congress.

Among those recently accepting office as Vice-Presidents of the Congress, we note Sir B. Walter Foster, President of the Council of the British Medical Association; Mr. Ernest Hart, Editor of the Journal of the British Medical Association; Sir Thomas Longmore, Surgeon-General (retired) of the British Army; Sir Joseph Fayrer, Surgeon-General of India; Mr. John Eric Erichsen, Sir Dyce Duckworth, and Mr. Jonathan Hutchinson, of England; Professor Dujardin-Beaumetz, M. Bouchut, M. Cadet de Gassicourt, and M. Grancher, of France; Professor Moncorvo, of Rio de Janeiro; M. Servais, of Belgium; Alfred Vogel, of Germany, with many others equally well known.

The Executive Committee will continue to prosecute its work, with the object of maintaining the honor of the profession in the United States, and making good the invitation given and accepted in 1884 at Copenhagen.

Arrangements are under consideration for reduced fares on steamships and railways, and in hotels.

